# The immunomodulator effect of *Stevia rebaudiana* Bertoni mediated by TNF‐α and IL‐1β in peripheral blood in diabetic rats

**DOI:** 10.1002/fsn3.4371

**Published:** 2024-07-29

**Authors:** Erhan Cebeci, Ertan Katirci, Mustafa Karhan, Emin Turkay Korgun

**Affiliations:** ^1^ Faculty of Medicine, Department of Histology and Embryology Akdeniz University Antalya Turkey; ^2^ Faculty of Medicine, Department of Histology and Embryology Ahi Evran University Kirsehir Turkey; ^3^ Faculty of Engineering, Department of Food Engineering Akdeniz University Antalya Turkey

**Keywords:** Asteraceae, cytokine, diabetes, lymphocyte, *Stevia rebaudiana* Bertoni

## Abstract

*Stevia rebaudiana* Bertoni, which is a medicinal plant used in the treatment of diabetes, was the focus of this study aiming to investigate its immunomodulatory properties in diabetes. To form the diabetes group, rats were injected intraperitoneally with STZ and rats with blood glucose levels above 200 mg/dL 2 days after STZ injection were included in the diabetes group. To form the stevia and diabetes + stevia groups, stevia was administered daily by gavage to both healthy and diabetic rats for 28 days. At the end of 28 days, the levels of interleukin‐1 beta and tumor necrosis factor‐alpha in the blood were measured by ELISA. CD3, CD4, and CD8 protein levels in the blood were determined by flow cytometry. Rat body weight increased in the diabetes +25 mg/kg bW stevia group compared with the diabetes group. Blood glucose levels were significantly decreased in the diabetes +25 mg/kg bW stevia group compared to the diabetes group (**p < .01). IL‐1β cytokine levels decreased significantly in the diabetes +25 mg/kg bW stevia group compared to the diabetes group (**p < .01). TNF‐α cytokine levels decreased significantly in the diabetes +25 mg/kg bW stevia group compared to the diabetes group (**p < .01). The amount of CD8 + T cells decreased significantly in the diabetes +25 mg/kg bW stevia group compared to the diabetes group (*p < .05). The stevia diet leads to a reduction in peripheral circulating cytotoxic T cells and proinflammatory cytokines interleukin‐1 beta and tumor necrosis factor‐alpha under hyperglycemic conditions.

## Graphical abstract

The study shows that stevia increases body weight and lowers blood glucose levels in diabetic rats. In addition, it was determined that stevia reduced the levels of proinflammatory cytokines (IL‐1β and TNF‐α) and decreased the amount of CD8+ T cells. These findings suggest that stevia may have immunomodulatory properties and may have a potential role in the treatment of diabetes.

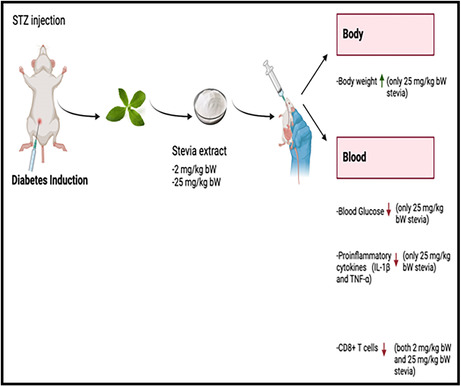

## 1 INTRODUCTION

As sugar, which we use as a sweetener in foods, causes diseases, artificial and natural sweeteners that can be an alternative to sugar have been started to be researched. Studies have shown that artificial sweeteners can increase calorie intake, body weight, and fat accumulation (Hampton, 2008; Swithers & Davidson, 2008). As a result, the use of natural sweeteners and their application in various clinical conditions has increased. One of the natural sweeteners is stevia plant. *Stevia rebaudiana* Bertoni is a plant belonging to the Asteraceae family that grows in South America. Although hundreds of times sweeter than sucrose, *Stevia rebaudiana* Bertoni has no calories (Nathalie et al., 2019). Recent research has delved into the health advantages of stevia that go beyond its sweetness. These studies have highlighted its hyperglycemic, anti‐inflammatory, and immune‐regulating properties (Boonkaewwan & Burodom, 2013; Jan et al., 2021). These properties of stevia have made it easier to treat diabetic conditions and have led to an increased variety of products to meet the needs of diabetics.

Diabetes mellitus (DM) is one of the most common endocrine diseases worldwide. There are two main types of diabetes: Type 1 diabetes (T1D) and Type 2 diabetes (T2D). T1D is caused by insufficient insulin production, while T2D is caused by reduced insulin sensitivity. T1D is characterized by the inability to produce sufficient insulin due to an autoimmune process that specifically targets and eliminates beta (β) cells within the pancreas (Clark et al., 2017). Studies have shown that CD4^+^ and CD8^+^ T lymphocytes play a key role in β cell destruction (Coppieters et al., 2012; Rodriguez‐Calvo et al., 2015; Sarikonda et al., 2014). In addition to these cells, β cell autoantigens, macrophages, dendritic cells, and B cells also contribute to this process. In T1D, many of the proteins targeted as autoantigens are found within the insulin secretory granule. However, the reason for the selection of secretory pathway proteins as antigens in T1D, as opposed to proteins from other cellular compartments or other macromolecules such as RNAs or carbohydrates, remains unknown. The first cell types to infiltrate the islets are macrophages and dendritic cells. Naive CD4+ T cells circulating in lymphoid organs and the blood recognize major histocompatibility complex (MHC) and β cell peptides presented by dendritic cells and macrophages infiltrating pancreatic islets. Interleukin‐12 (IL‐12) released by macrophages and dendritic cells activate these CD4+ T cells. Meanwhile, CD8+ T cells specific for β cell antigens are activated by interleukin‐2 (IL‐2) produced by activated T helper 1 (Th1) CD4+ T cells. This leads to their differentiation into cytotoxic T cells and their infiltration into the islets. In addition, β cells can be damaged by soluble mediators such as granzymes and perforin released by CD8+ cytotoxic T cells, and by cytokines and reactive oxygen molecules released by activated macrophages in the islets. Thus, activated macrophages, Th1 CD4+ T cells and β cell cytotoxic CD8+ T cells, act synergistically to cause autoimmune T1D (Yoon & Jun, 2005).

Overall, researchers worldwide agree on the anti‐diabetic effects of *Stevia rebaudiana* Bertoni. In some studies of STZ or Alloxan‐induced diabetic rats, stevia extracts administered by different methods have been shown to reduce blood glucose levels and have anti‐hyperglycemic effects. Oral stevia extract caused a time‐dependent decrease in blood glucose levels in diabetic rats, anti‐hyperglycemic effects, and a reduction in hepatic gluconeogenesis in diabetes‐induced mice (Hossain et al., 2011; Misra et al., 2011). A study by Bessler et al. investigated the effects of three different sweeteners, namely Sweet'N Low, Splenda, and Stevia, on cytokine expression in peripheral blood mononuclear cells (PBMCs). This study showed that the potential of stevia as cancer preventive agents is indicated due to their ability to inhibit pro‐inflammatory cytokines (IL‐1β, TNFα, and IL‐6) and increase the release of anti‐inflammatory cytokines (IL‐10 and IL‐1ra) (Bessler & Djaldetti, 2019). In another study investigating the effect of different sweeteners (Sucrose, Suclarose and Stevia) on cytokines secreted by CD4+ T cells isolated from Peyer's patches, stevia was found to reduce interferon gamma (IFN‐γ) release (Rosales‐Gómez et al., 2018).

T1D is an autoimmune disease characterized by excessive inflammation. CD4+ and CD8+ T lymphocytes have been identified as key players in the pathogenesis of T1D. In the literature, the effect of stevia on the excessive inflammation observed in T1D and its protective effects on T cell‐induced β cell apoptosis remain unclear. Furthermore, the effect of stevia on proinflammatory cytokines, which are increased due to excessive inflammation, is not clear. Therefore, the aim of this study was to evaluate the effects of stevia extract on the number of T lymphocytes and the release of inflammatory cytokines such as TNF‐α and IL‐1β in the peripheral blood of normal and STZ‐induced diabetic animals. This study specifically focuses on the effects of stevia in the context of T1D.

## 2 MATERIALS AND METHODS

### 2.1 Experimental groups

Female Wistar rats were obtained from the Animal Experiment Unit at the University of Akdeniz (date of approval: February 10, 2020 and approval number: 2020.02.006) aged 8 weeks and weighing 150–250 g. Wistar rats were kept under a 12 h light–dark cycle and were fed a standard diet. Animal experiments were carried out in accordance with ARRIVE guidelines (Kilkenny et al., 2010). Rats were randomly split into six groups with six rats for each group. Rats were injected intraperitoneally with a single dose of 50 mg/kg bW STZ (Deeds et al., 2011). Blood glucose levels were measured 2 days after STZ injection. Only rats with blood glucose levels above 200 mg/dL were considered diabetic and included in the study. Stevia was administered by gavage at 2 and 25 mg/kg bW daily for 28 days in the stevia and diabetes + stevia groups.

### 2.2 Extraction of steviol glycosides from dry stevia leaves

Dry stevia leaves were separated from foreign materials and ground by a laboratory‐type blender (Waring, USA). Ground stevia leaves were mixed with distilled water at a ratio of 1:10 (ground leaf: water) for extraction and were incubated in a water bath for 30 min at 40°C. Afterward, centrifugation was applied at 2900× *g* force (750 mL tubes) at 25°C for 30 min and filter paper was used to separate the stevia leaves from the extract (Kulcan & Karhan, 2020). Before the membrane process application, stevioside and reb A were analyzed. Stevia extracts were diluted 1:20 with ultrapure water and analyzed by high‐performance liquid chromatography to determine the reb A and stevioside content (HPLC). *Membrane process* diafiltration and nanofiltration were applied to extracts for concentration, removing the bitter taste and herbaceous smell. 100 and 5 kDa polyethersulfone (PESU) membranes were used for diafiltration and nanofiltration processes (Yildiz & Karhan, 2021). Stevia plant under study is endotoxin free.

### 2.3 Flow cytometry

Peripheral blood samples were collected from the abdominal aorta of rats after 28 days of stevia diet administration by gavage. For flow cytometry, 1 mL of blood was collected into purple‐capped EDTA anticoagulant blood collection tubes (BD Vacutainer K2EDTA). For each sample, 100 μL of blood was collected into 1.5 mL sterile Eppendorf tubes. CD3 FITC‐conjugated (BIOLEGEND‐201403), CD4 PE‐conjugated (BIOLEGEND‐201507), and CD8 PerCP (BIOLEGEND‐201712) antibodies (1 μL) were added to the blood samples. The samples were incubated for 30 min in the dark at room temperature. FACS Lysing Solution (900 μL) was added to Eppendorf tubes. The mixture was vortexed and incubated on ice for 15 min. Centrifugation was performed at 400 *g* for 5 min. The supernatant was removed. 1 mL of PBS‐NaN3 was added and centrifuged at 400 *g* for 5 min. The supernatant was removed. 1 mL of PBS‐NaN3 was added and centrifuged at 400 *g* for 5 min. The supernatant was removed. The pellet was dissolved in 400 μL of PBS‐NaN_3_ and measurements and analyses were performed using CytExpert software in the flow cytometer.

### 2.4 Elisa

Peripheral blood samples were collected from the abdominal aorta of rats after 28 days of stevia diet administration by gavage. For the ELISA, blood samples collected from rats were centrifuged at 5000 rpm for 10 min. The remaining serum was placed in an Eppendorf tube. The assay was performed to the protocols of the Rat IL‐1β PicoKine EK0393 and Rat TNF‐α PicoKine EK0526 ELISA Kits.

### 2.5 Statistical analysis

The means, standard deviations, and medians of the experimental data were calculated using descriptive statistics. Data are presented as the mean ± SEM. Quantitative data were tested for normality using the Shapiro–Wilk normality test. Data were analyzed using paired t‐test. Data were analyzed using one‐way ANOVA followed by Holm‐Sidak post hoc test for data passing the normality test; otherwise, the Kruskal–Wallis test was used followed by Dunn's test (GraphPad Prism 9). Differences between groups were considered statistically significant at *p* < .05.

## 3 RESULTS

### 3.1 Rat weights

**FIGURE 1:**
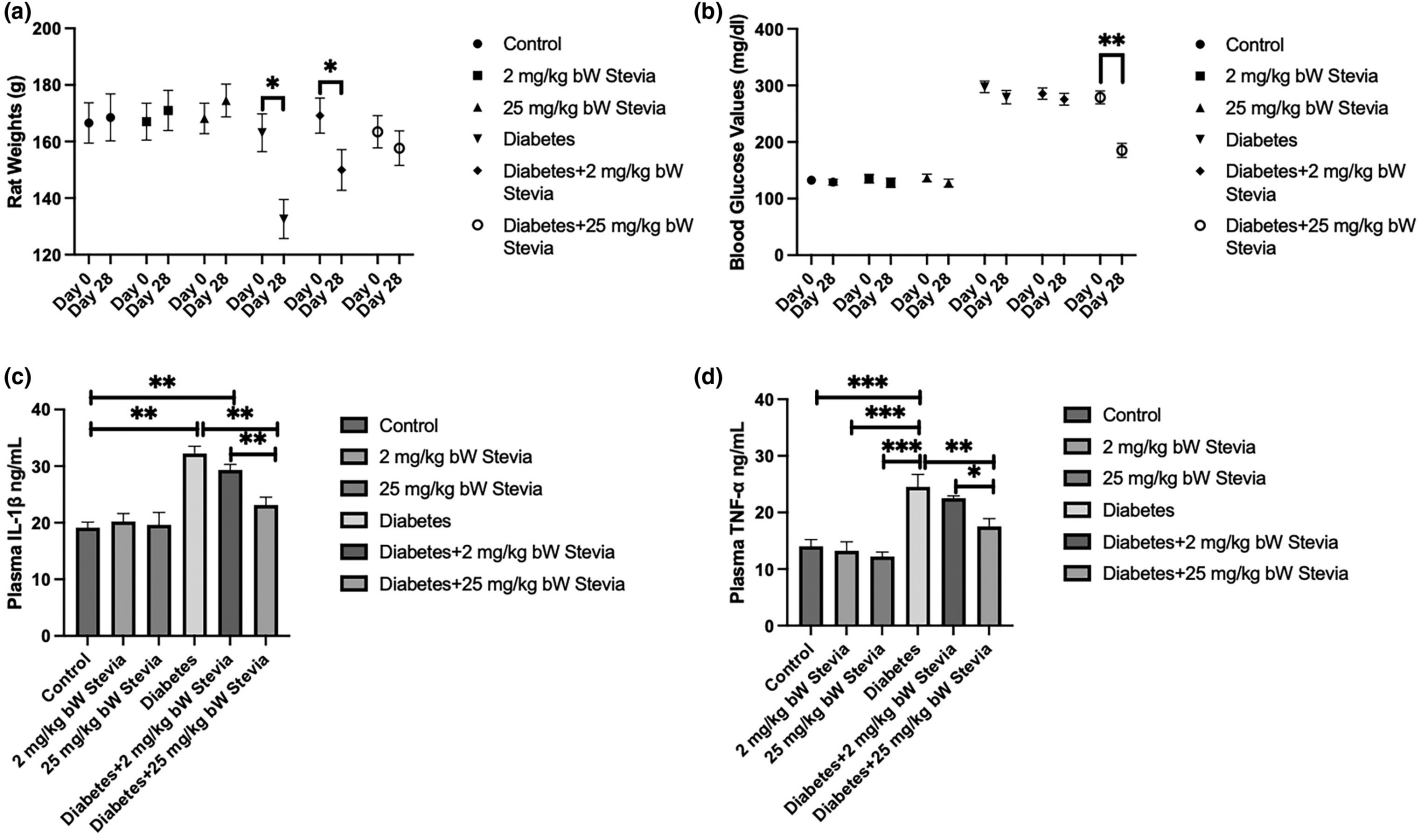
Comparison of rat weights, blood glucose levels, TNF‐α, and IL‐1β protein levels in control, diabetes, and stevia groups. (a) Comparison of rat weights, (b) Comparison of blood glucose values, (c) Comparison of IL‐1β protein level, (d) Comparison of TNF‐α protein level of control, 2 mg/kg bW stevia, 25 mg/kg bW stevia, diabetes, diabetes +2 mg/kg bW stevia, and diabetes +25 mg/kg bW stevia groups on day 0 and 28. Data are presented as mean ± SEM. (a, b) Statistical significance was determined using paired *t*‐test. (c, d) Statistical significance was determined using one‐way ANOVA followed by Tukey's post‐hoc test. * indicates *p* < .05, ** indicates *p* < .01, *** indicates *p* < .001. *n* = 6. The experiment was repeated three times independently.

The weights of rats in all groups were measured on days 0 and 28. In the diabetes and diabetes+2 mg/kg bW stevia groups, rat weights decreased significantly on the 28th day compared to the 0th day (*p < .05). The weights of rats in the diabetes +25 mg/kg stevia group did not change between day 0 and day 28 (*p* > .05). This suggests that stevia may reduce diabetes‐induced weight loss in a dose‐dependent manner. (Figure 1a).

### 3.2 Blood glucose measurements

The blood glucose values of rats in all groups on days 0 and 28 were evaluated. The administration of STZ injection was carried out on the diabetic groups, and two days following the initial administration, the blood sugar levels were found to be 200 mg/dL higher. The blood glucose value showed a statistically substantial increase in the groups with diabetes mellitus with STZ. At the end of 28 days, the highest blood glucose levels were observed in the diabetic group. In the diabetes +2 mg/kg bW stevia group, no significant difference was detected at the end of 28 days compared to day 0. In the diabetes +25 mg/kg bW stevia group, a significant decrease was detected at the end of 28 days compared to day 0 (**p < .01). These results suggest that stevia can lower blood glucose in diabetic conditions and that this effect is dose‐dependent. On the other hand, no significant changes were detected in the non‐diabetic groups (Figure 1b).

### 3.3 Elisa

According to the blood serum IL‐1β results, the highest amount of IL‐1β was observed in the diabetic group on the 28th day among the groups. IL‐1β levels were statistically significantly higher in diabetes and diabetes +2 mg/kg bW stevia group than in control, 2 mg/kg bW stevia, 25 mg/kg bW stevia, and diabetes +25 mg/kg bW stevia groups (**p < .01). The diabetes +25 mg/kg bW stevia group had significantly lower protein content than the diabetes and diabetes +2 mg/kg bW stevia groups (**p < .01) (Figure 1c).

According to the blood serum TNF‐α results, the highest amount of TNF‐α level was observed in the diabetic group. TNF‐α levels were statistically significantly higher in diabetic group than in control, 2 mg/kg bW stevia, 25 mg/kg bW stevia, and diabetes +25 mg/kg bW stevia groups (***p < .001). The diabetes +25 mg/kg bW stevia group had significantly lower protein content than the diabetes and diabetes +2 mg/kg bW stevia groups (respectively **p < .01 and *p < .05) (Figure 1d).

### 3.4 Flow cytometry

**FIGURE 2:**
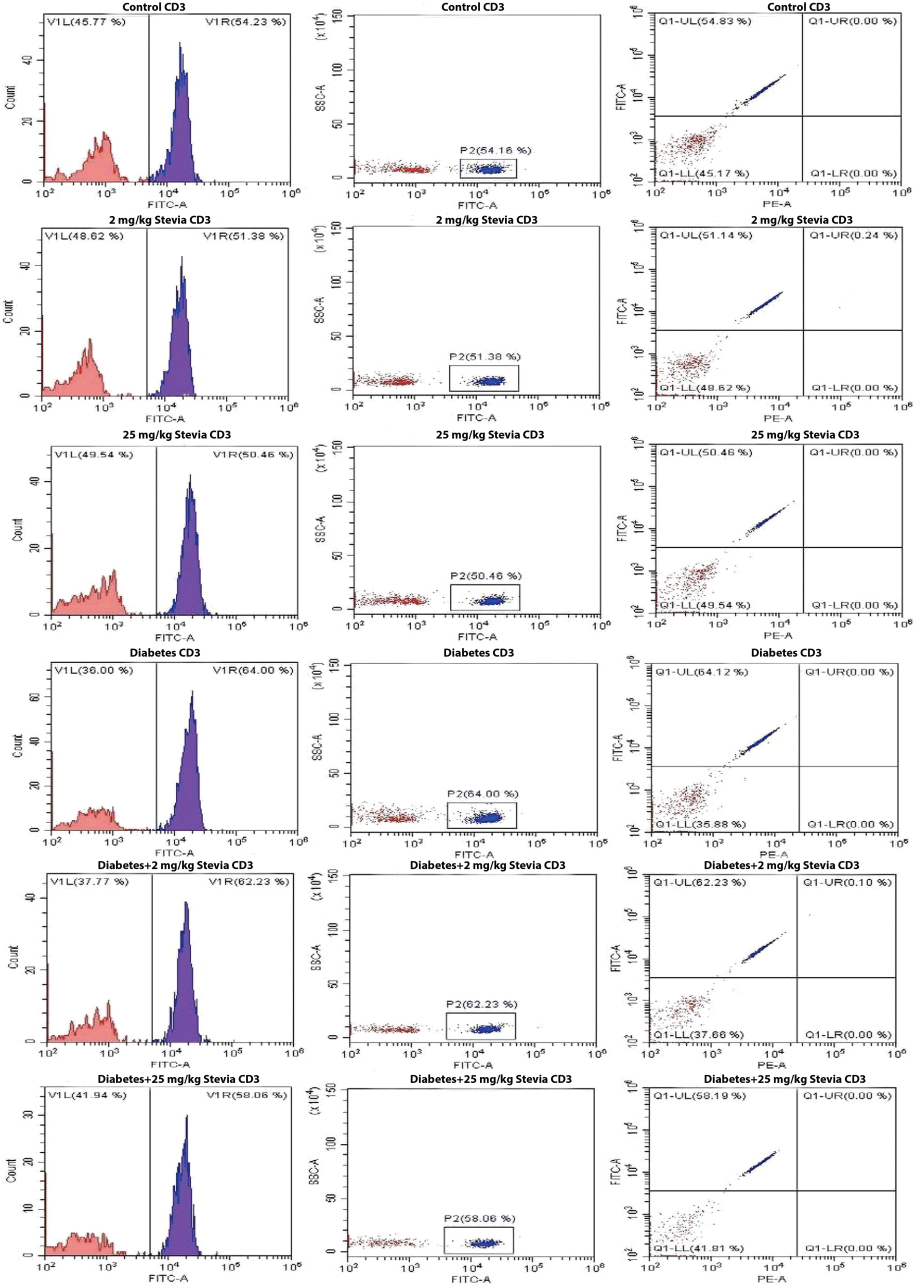
Quantification of CD3+ T cells in control, 2 mg/kg bW stevia, 25 mg/kg bW stevia, diabetes, diabetes +2 mg/kg bW stevia, and diabetes +25 mg/kg bW stevia groups. *n* = 6. The experiment was repeated three times independently.

**FIGURE 3:**
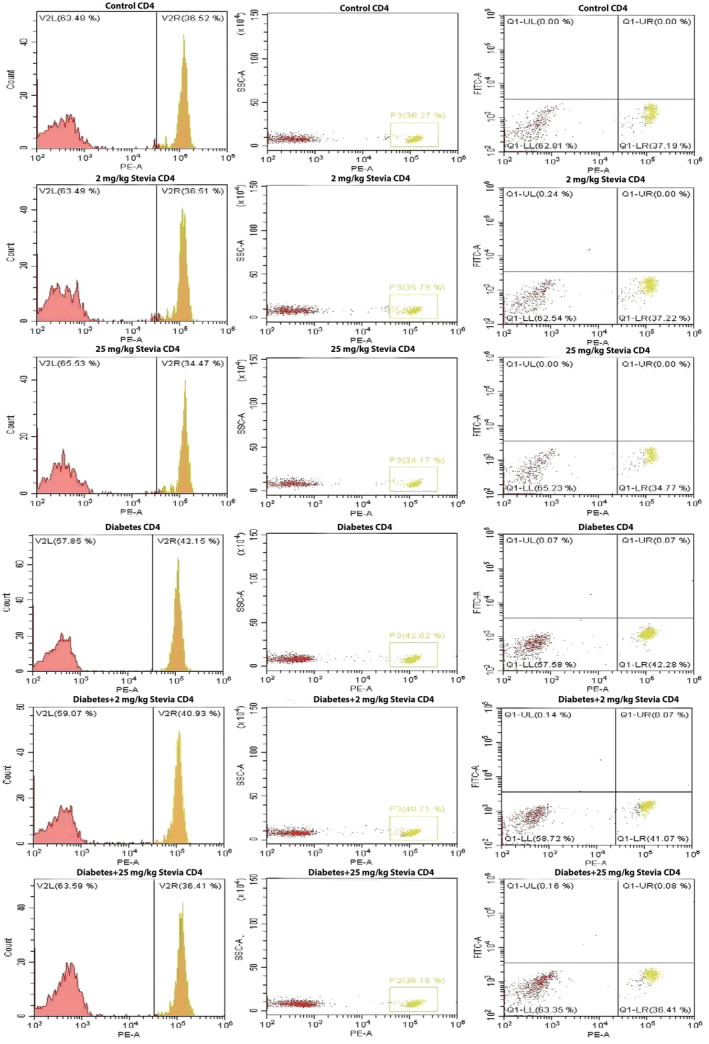
Quantification of CD4+ T cells in control, 2 mg/kg bW stevia, 25 mg/kg bW stevia, diabetes, diabetes +2 mg/kg bW stevia, and diabetes +25 mg/kg bW stevia groups. *n* = 6. The experiment was repeated three times independently.

**FIGURE 4:**
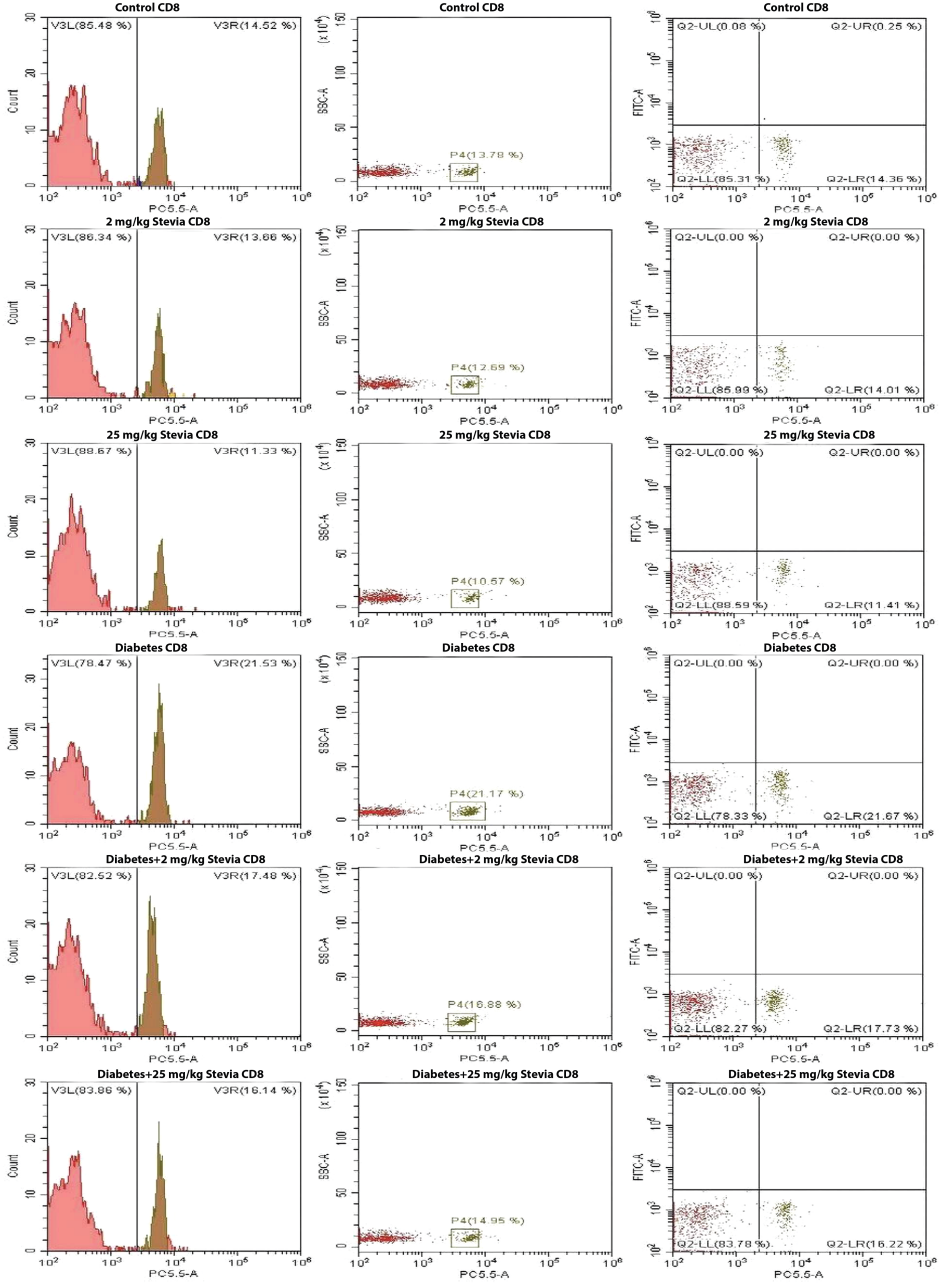
Quantification of CD8+ T cells in control, 2 mg/kg bW stevia, 25 mg/kg bW stevia, diabetes, diabetes +2 mg/kg bW stevia, and diabetes +25 mg/kg bW stevia groups. *n* = 6. The experiment was repeated three times independently.

**FIGURE 5:**
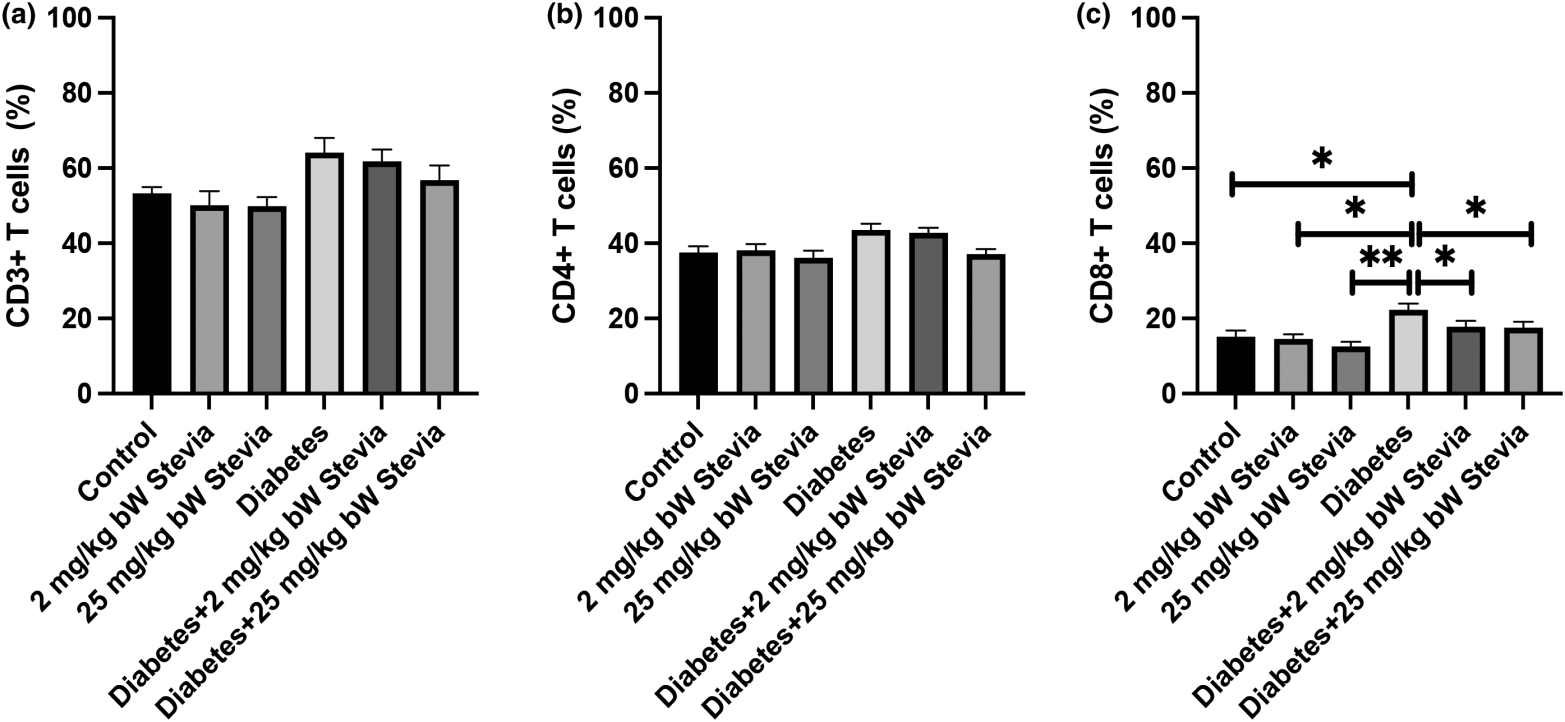
Comparison of (a) CD3+ T Cells, (b) CD4+ T Cells, (c) CD8+ T Cells among in control, 2 mg/kg bW stevia, 25 mg/kg bW stevia, diabetes, diabetes +2 mg/kg bW stevia, and diabetes +25 mg/kg bW stevia groups. Data are presented as mean ± SEM. Statistical significance was determined using a one‐way ANOVA followed by Tukey's post‐hoc test. * indicates *p* < .05, ** indicates *p* < .01. *n* = 6. The experiment was repeated three times independently.

Total T lymphocyte, CD4+, and CD8+ T lymphocyte counts in all groups were determined using flow cytometry (Figures 2, 3, 4) (Table 1). When the blood samples of the groups were collected and the total T lymphocyte cell counts were compared, it was determined that the number of T cells in the diabetic group was higher than that in the other groups, but this increase was not significant (Figure 5a). Cell counts of CD4+ T lymphocytes were evaluated between control, 2 mg/kg bW stevia, 25 mg/kg bW stevia, diabetes, diabetes +2 mg/kg bW Stevia, and diabetes +25 mg/kg bW stevia groups. Although the CD4+ cell count was highest in the diabetes group, this increase was not significant (Figure 5b). CD8+ T lymphocyte counts were determined for all groups. When the groups were compared, the highest CD8+ T lymphocyte count was found in the diabetes group. There was no statistically significant difference among control, 2 mg/kg bW stevia, and 25 mg/kg bW stevia groups. Similarly, statistical significance was not determined between diabetes +2 mg/kg bW stevia and diabetes +25 mg/kg bW groups. When the CD8 protein level in the diabetes +2 mg/kg bW stevia and diabetes +25 mg/kg bW stevia groups were compared with the diabetes group, the CD8 amount of the diabetes group was found to be significantly higher (*p < .05) (Figure 5c).

**TABLE 1:** Percentage and SEM values of CD3, CD4, and CD8 cell counts of control, 2 mg/kg Stevia, 25 mg/kg Stevia, Diabetes, Diabetes +2 mg/kg Stevia, and Diabetes +25 mg/kg Stevia groups on day 28.

	CD3 (%)	CD4 (%)	CD8 (%)
Control	53.33 ± 1.59	37.5 ± 1.70	15.16 ± 1.63
2 mg/kg stevia	50.16 ± 3.71	38.16 ± 1.67	14.5 ± 1.29
25 mg/kg stevia	49.83 ± 2.38	36.16 ± 1.91	12.5 ± 1.25
Diabetes	64.16 ± 3.91	43.5 ± 1.70	22.33 ± 1.63
Diabetes + 2 mg/kg bW stevia	61.83 ± 3.13	42.83 ± 1.29	17.83 ± 1.57
Diabetes + 25 mg/kg bW stevia	56.83 ± 3.89	37.16 ± 1.31	17.5 ± 1.62

## 4 DISCUSSION

The objective of this study is to investigate the protective effect of stevia against T cell‐mediated β cell destruction in diabetic rats and to explore its impact on proinflammatory cytokines that are elevated as a result of increased inflammation. Rat weights were evaluated in experimental groups. The diabetes +25 mg/kg bW stevia group showed a rise, in body weight unlike the diabetes +2 mg/kg bW stevia group, when compared to the diabetes group. These findings are consistent with previous studies (Das et al., 2017; Rosales‐Gómez et al., 2018). This suggests that stevia might offer protection against weight loss linked to diabetes in a way that depends on the dosage. Rat blood glucose measurements were evaluated in experimental groups. The diabetes +25 mg/kg bW stevia group showed a significant decrease in blood glucose measurements compared to the diabetes group, in contrast to the diabetes +2 mg/kg bW stevia group. In many previous studies, blood glucose levels in the group of rats with diabetes mellitus treated with stevia were found to be statistically lower compared to the diabetic control group that did not receive stevia (Ahmad & Ahmad, 2018; Assi et al., 2020; Das et al., 2017). In another study, blood glucose levels were similar in the diabetes and diabetes + stevioside groups (Katirci et al., 2023). In the present study, stevia may reduce the rise in blood glucose levels caused by diabetes by increasing insulin secretion in β cells (Chowdhury et al., 2022).

According to the results of IL‐1β ELISA in the serum, the highest amount of IL‐1β was detected in the diabetes group. A statistically significant decrease was detected in the diabetes +25 mg/kg bW stevia group compared to the diabetes group (**p < .01). IL‐1β level was statistically significantly higher in diabetes and diabetes +2 mg/kg bW stevia groups compared to control, 2 mg/kg bW stevia, 25 mg/kg bW stevia, and diabetes +25 mg/kg bW stevia groups. According to our TNF‐α ELISA results, the highest amount of TNF‐α in the serum was determined in the diabetes group. A significant decrease was detected in the diabetes +25 mg/kg bW stevia group compared to the diabetes group. TNF‐α level was statistically significantly higher in diabetes and diabetes +2 mg/kg bW stevia groups compared to control, 2 mg/kg bW stevia, 25 mg/kg bW stevia, and diabetes +25 mg/kg bW stevia groups. Our study and other studies in the literature show that stevia regulates the release of proinflammatory cytokines in diabetic rats (Bessler & Djaldetti, 2019; Francisco et al., 2016; Ramos‐Tovar et al., 2018). IL‐1 β and TNF‐ α cytokines are key cytokines in pancreatic beta cell inflammation in T1D (Coomans de Brachène et al., 2024). Our results show that stevia reduces peripheral circulating levels of proinflammatory cytokines that cause β‐cell loss in diabetic rats. Therefore, stevia may alleviate the inflammation involved in β‐cell destruction by reducing the levels of proinflammatory cytokines.

According to our flow cytometry results, the amount of CD3^+^ T cells and CD4^+^ T cells were the highest in the diabetes group. The amount of CD8^+^ was found to be statistically at the highest level in the diabetes group. In a study by Walker et al., it was reported that CD8+ T cells are at higher levels in diabetic patients than in healthy people (Walker & von Herrath, 2016). When our research is evaluated together with other studies in the literature, it shows that stevia has a negative effect on the number of cytotoxic T cells. Our findings show that stevia may reduce the peripheral circulating number of CD8^+^ T cells that cause β‐cell loss in diabetic rats. Considering the effects of IL‐1β and TNF‐α on T cell activation and proliferation, these results indicate that stevia's effects on total T cells, helper T cells, and cytotoxic T cells warrant further investigation (Ben‐Sasson et al., 2013; Mehta et al., 2018). Its effects on TNF‐α and IL‐1β cytokines show a correlation.

## 5 CONCLUSION

Stevia diet causes a decrease in peripheral circulating cytotoxic T cells and proinflammatory cytokines TNF‐alpha and IL‐1β under hyperglycemic conditions. Thus, it may regulate the inflammation process in diabetes by reducing them.

## AUTHOR CONTRIBUTIONS

**Erhan Cebeci:** Formal analysis (lead); investigation (lead); validation (lead); visualization (lead); writing – original draft (lead). **Ertan Katirci:** Writing – review and editing (supporting). **Mustafa Karhan:** Methodology (supporting). **Emin Turkay Korgun:** Conceptualization (lead); funding acquisition (lead); methodology (lead); project administration (lead); supervision (lead); writing – review and editing (lead).

## FUNDING INFORMATION

This study was supported by Akdeniz University Scientific Research Projects Coordination Unit. Project number: TYL‐2020‐5495.

## CONFLICT OF INTEREST STATEMENT

The authors have no competing interests to declare that are relevant to the content of this article.

## Data Availability

Data are available on request from the authors.
